# Fast inexact mapping using advanced tree exploration on backward search methods

**DOI:** 10.1186/s12859-014-0438-3

**Published:** 2015-01-28

**Authors:** José Salavert, Andrés Tomás, Joaquín Tárraga, Ignacio Medina, Joaquín Dopazo, Ignacio Blanquer

**Affiliations:** 1 GRyCAP department of I3M, Universitat Politècnica de València, Building 8B, Camino de vera s/n, Valencia, 46022 Spain; 2 Bioinformatics department of Centro de Investigación Príncipe Felipe, Autopista del Saler 16, Valencia, 46012 Spain

**Keywords:** Sequence mapping, Burrows-wheeler, FM-Index, Suffix array

## Abstract

**Background:**

Short sequence mapping methods for Next Generation Sequencing consist on a combination of seeding techniques followed by local alignment based on dynamic programming approaches. Most seeding algorithms are based on backward search alignment, using the Burrows Wheeler Transform, the Ferragina and Manzini Index or Suffix Arrays. All these backward search algorithms have excellent performance, but their computational cost highly increases when allowing errors. In this paper, we discuss an inexact mapping algorithm based on pruning strategies for search tree exploration over genomic data.

**Results:**

The proposed algorithm achieves a 13x speed-up over similar algorithms when allowing 6 base errors, including insertions, deletions and mismatches. This algorithm can deal with 400 bps reads with up to 9 errors in a high quality Illumina dataset. In this example, the algorithm works as a preprocessor that reduces by 55% the number of reads to be aligned. Depending on the aligner the overall execution time is reduced between 20–40%.

**Conclusions:**

Although not intended as a complete sequence mapping tool, the proposed algorithm could be used as a preprocessing step to modern sequence mappers. This step significantly reduces the number reads to be aligned, accelerating overall alignment time. Furthermore, this algorithm could be used for accelerating the seeding step of already available sequence mappers. In addition, an out-of-core index has been implemented for working with large genomes on systems without expensive memory configurations.

## Background

In the field of bioinformatics, the term alignment [1] refers to identifying similar areas between chains of DNA, RNA or protein primary structures. The alignment is the first step in most studies of functional or evolutionary relationships between genes or proteins.

With the advent of high-throughput sequencing techniques, a topic frequently addressed is the mapping of short DNA sequences on a consensus reference genome. In this case, differences between reads and the reference appear, due to the natural genetic variability or failures in the sequence digitalisation phase. For this reason, a mapping algorithm must allow a certain number of errors, guaranteeing that sequences slightly different to the reference will be mapped.

Several inexact alignment solutions available in the literature focus on dynamic programming approaches, like the *Smith-Waterman* Algorithm [2,3] (SW) or the *Hidden Markov Models* [4] (HMM). However, their computational complexity depends on the length of the read multiplied by the length of the reference genome.

A different approach to sequence alignment are backward search techniques based on the *Burrows Wheeler Transform* (BWT). Its main advantage over the dynamic programming approaches is that its computational complexity depends only on the length of the read. However, backward search techniques need to initially generate an index of the reference using the *Ferragina and Manzini Index* [5] (FM-Index).

The BWT has been originally used in data compression techniques [6,7], but the FM-Index [8] allowed the design of recursive backwards searching algorithms for inexact mapping [9]. This is the case of BWA-backtrack [9], SOAP2 [10], and Bowtie 1 [11]. More recently, SOAP3-dp [12], CUSHAW2 [13], Barracuda [14] and our previous work [15] support GPU computing. FPGA implementations [16] are also available.

Additionally, other prefix search techniques are based on *Suffix array* [17] (SA) and enhanced SA [18] theory with applications to bioinformatics. Specifically, essaMEM [19] and Psi-Ra [20] are based on sparse SA. Backward search methods can also be applied to SA in a similar fashion as the FM-Index [21]. The computational cost of prefix search methods depends on the prefix length and a constant value that can be improved depending on the data structures.

However, backward search methods performance decreases with the number of errors allowed during the alignment. Backward search inexact mapping combines an exact search procedure with a search tree exploration routine that checks all the possible solutions within the number of errors allowed. All practical implementations avoid this exponential cost using pruning or greedy strategies [9,11,22,23].

For this reason, backward search techniques are employed to locate small segments of the reads (seeds) in the genome, revealing alignment candidate areas. Subsequently, a local alignment algorithm maps the read against the highlighted area only, reducing the computational cost. BWA [24], Bowtie 2 [25] and SeqAlto [26] combine FM-Index multi-seed preprocessing with dynamic programming methods. SSAHA2 [27] and GEM [28] locate k-mers to be used as prefixes that are also explored using dynamic programming approaches.

Although these combined methods are faster than performing a full dynamic programming analysis and its sensitivity is undisputed when dealing with long reads and big gaps, it is still desirable to further improve inexact backward search mapping methods.

The algorithm described in this paper is intended as an extra preprocessing step before the seed location phase of current sequence mappers. Our main objective is to reduce the number of read locations to be analysed by more expensive combined strategies. We need a more efficient algorithm capable of dealing with longer reads and able to reuse part of the backward search preprocessing to accelerate the seeding phase. We demonstrate that these goals can be achieved by improving previous research [9,23].

This algorithm improves the inexact mapping of short reads with several bounding strategies suited for genomic data. We support all type of errors (insertions, deletions and mismatches) in all the positions of the read, while other tools impose limits due to the growth of the search tree. Moreover, none of the bounding strategies described in this article decrease the sensitivity of the search, returning all the existing mappings given a maximum number of errors. Experimental results show a great increase in the performance over similar approaches, proving its viability with longer reads.

The work described here is based on replaceable components, providing the necessary interfaces to be compatible with any tool using a backward search method. In this study, we used both our own backward search implementation of the FM-Index and the implementation for DNA from *csalib* [29], this library implements several backward search methods based on either the FM-Index or SA [21]. Using *csalib*, data structures are not loaded into main memory [30], but accessed from disk by demand using *mmap*. This property may be useful in memory demanding tasks, like mapping against big genomes.

## Methods

We divide the methods specification into five sections. First of all, we introduce the backward search method on top of which the algorithm is executed. Secondly, we describe the compression mechanisms of the data structures, due to its potential effect in the performance. Thirdly, we present a simplified version of the algorithm as a start point to include the different pruning techniques. Fourthly, we outline the complete search tree exploration algorithm and the remaining pruning strategies. Finally, we detail the hardware used during the benchmarks and the properties of the datasets.

### Backward search

The backward search method used in the benchmarks is based on our own implementation of the FM-Index data structures, here we present a brief introduction to the FM-Index search theory.

Let *A*={*A*,*C*,*G*,*T*} be an alphabet and $ a symbol not included in *A*, with less lexicographic value than all the symbols in *A*. Let *X*= “AGGAGC$” be the reference genome, a string of *A* terminated with the $ symbol.

The BWT can be obtained by sorting the suffixes of *X* (equation 1) with specific suffix array sorting Algorithms [31]. However, we employ a more recent approach that computes the BWT directly [32], without storing the full SA positions into memory.

(1) $$ \left(\begin{array}{ccccccc} A & G & G & A & G & C & \$ \\ G & G & A & G & C & \$ & A \\ G & A & G & C & \$ & A & G \\ A & G & C & \$ & A & G & G \\ G & C & \$ & A & G & G & A \\ C & \$ & A & G & G & A & G \\ \$ & A & G & G & A & G & C \end{array} \right) \begin{array}{c} 0 \\ 1 \\ 2 \\ 3 \\ 4 \\ 5 \\ 6 \end{array}  $$

After this preprocessing, *B*=[ *C*,*G*,*$*,*G*,*G*,*A*,*A*] contains the BWT of *X* (equation 2).

(2) $$ \left(\begin{array}{cccccc|c} \$ & A & G & G & A & G & C \\ A & G & C & \$ & A & G & G \\ A & G & G & A & G & C & \$ \\ C & \$ & A & G & G & A & G \\ G & A & G & C & \$ & A & G \\ G & C & \$ & A & G & G & A \\ G & G & A & G & C & \$ & A \end{array} \right) \begin{array}{c} 6 \\ 3 \\ 0 \\ 5 \\ 2 \\ 4 \\ 1 \end{array}  $$

The backward search method is based on the FM-Index [8] data structures. Let *a*∈*A*: *C*(*a*) be the number of symbols in *B* lexicographically smaller than *a* (equation 3) and let *O*(*a*,*i*) be the number of occurrences of symbol *a* in *B*[ 0:*i*−1] (equation 4, the first column is -1 and is always 0).

(3) $$ C = \left(\begin{array}{cccc} 0 & 2 & 3 & 6 \end{array} \right)  $$

(4) $$ O = \left(\begin{array}{cccccccc} 0 & 0 & 0 & 0 & 0 & 0 & 1 & 2 \\ 0 & 1 & 1 & 1 & 1 & 1 & 1 & 1 \\ 0 & 0 & 1 & 1 & 2 & 3 & 3 & 3 \\ 0 & 0 & 0 & 0 & 0 & 0 & 0 & 0 \end{array} \right) \begin{array}{c} A \\ C \\ G \\ T \end{array}  $$

Vector *S*=[ 6,3,0,5,2,4,1] is the numerical representation of the SA, with the permutation of each suffix (equation 2). Additionally, we define *R*=[ 2,6,4,1,5,3,0] as the *inverse of the SA* (ISA), satisfying *R*[ *S*[ *i*]]=*i*. *S* and *R* can be reconstructed from the FM-Index and the position of the $ symbol in *B* [33] (see section *Data structures compression*).

We also define *B*_*r*_, *O*_*r*_, *S*_*r*_ and *R*_*r*_ as the data structures of the reversed reference text X _*r*_. The reverse index allows to change the direction of the analysis during the search, but increases the memory requirements. Bidirectional methods [23] solve this issue but may not be efficient in all cases, see section *breadth-first exploration* for a more detailed discussion.

The occurrences of a string *W* in *X* constitute a contiguous interval [ *k*,*l*] in the sorted SA. *S*[ *k*…*l*] contains the positions of all the suffixes of *X* that start with *W*.

Backward search methods iteratively approximate the [ *k*,*l*] interval of a string *W* (Algorithm 1). The initial values are *k*=0 and *l*=*O*.*c* *o* *l*−2=|*S*|−1. On each **search_iteration** a symbol of *W* is analysed obtaining an equal or narrower [ *k*,*l*] interval for the larger substring. At the end, if *k*≤*l* string *W* belongs to *X*.



We return the result in variable *r* using a special notation ([ *k*,*l*] at *i* with [ ]), this means that we return interval [ *k*,*l*], pointing to the symbol of *W* at position *i* (where the search stopped) and setting and empty error list (this is exact search). The notation of the error list is described in the *Search tree exploration prototype* section.

In the case of the FM-Index **search_iteration**←**fm_iteration** (Algorithm 2).



Any backward search runtime providing an implementation of the **search_iteration** function and wrappers to access the SA and ISA will be compatible with the algorithm described in this paper.

### Data structures compression

Matrix *O* has a size that depends on the length of the genome times the size of the alphabet. Each element in *O* is a long integer, so therefore, *O* is a huge matrix to keep in memory. We compress matrix *O*, with *n* columns, into two matrices.

Let *O*_*count*_ be a matrix whose elements are bit vectors of size *w*, such vectors can be stored as integer values. The size of each row is *n*/*w* elements of *w* bits. If the i-th bit of a row is set to 1 this indicates that in the i-th position of *B* a nucleotide corresponding to the current row symbol appears.

Let *O*_*disp*_ be a matrix of integers with size *n*/*w*, where each element corresponds to a bit vector in *O*_*count*_. *O*_*disp*_[ *a*,*k*] contains the number of nucleotides of type *a*∈[ *A*,*C*,*G*,*T*] before the first bit of *O*_*count*_[ *a*,*k*].

To obtain *O*[ *a*,*i*] we add the number of nucleotides stored in *O*_*disp*_[ *a*,*i*/*w*] to the total count of 1 in *O*_*count*_[ *a*,*i*/*w*] until the last bit corresponding to *B*[ 0..*i*]. The *bit-count* operation is implemented at hardware level in many CPU and GPU, being a fast implementation.

Using this compression matrix O of *homo sapiens* requires 2 gigabytes with *w*=64, which is the typical CPU word size.

Let *S*, *R*, *Scomp* and *Rcomp* be integer vectors. Let *n* be the size of vector *S* and *R*, then *Scomp* and *Rcomp* size is *n*/*r* where *r* is the compression ratio. Each element of *Scomp* and *Rcomp* satisfies *S* *c* *o* *m* *p*[ *k*]=*S*[ *k*∗*r*] and *R* *c* *o* *m* *p*[ *k*]=*R*[ *k*∗*r*] respectively. In equation 5 we define *Ψ* ^−1^ as the inverse compressed suffix array, (*Ψ* ^−1^)^(*j*)^ denotes applying *Ψ* ^−1^ for *j* times.

In equation 6 we reconstruct *S* from *Scomp* [33]. In order to obtain *S*[*k*] we repeatedly apply *Ψ* ^−1^ until we reach some *j* value which satisfies that *S*[(*Ψ* ^−1^)^(*j*)^(*k*)] is a multiple of *r* stored in *Scomp*.

(5) $$\begin{array}{@{}rcl@{}} & \Psi^{-1}(i) = C(B[i])+O(B[\!i],i+1)  \end{array} $$

(6) $$\begin{array}{@{}rcl@{}} & S[\!k] = S\left[\left(\Psi^{-1}\right)^{(j)}(k)\right]+j  \end{array} $$

(7) $$\begin{array}{@{}rcl@{}} & R[\!k] = \left(\Psi^{-1}\right)^{(j')}\left(R\left[k+j'\right]\right)  \end{array} $$

(8) $$\begin{array}{@{}rcl@{}} & j' = (r - (k \bmod r)) \bmod r  \end{array} $$

In equation 7 we reconstruct *R* from *Rcomp* following similar principles. To obtain *R*[ *k*] we first calculate *j* ^′^ with equation 8. With *j* ^′^, we are able to obtain *R*(*k*+*j* ^′^), as it is a multiple of *r* stored in *Rcomp*. After that, we apply *Ψ* ^−1^ for *j* ^′^ repeated times to obtain *R*[ *k*].

Vectors *S* and *R* need 0.75 GB each for the *homo sapiens* genome with a compression ratio of *r*=16. There are more advanced compression techniques but our approach is a good balance between memory and speed in a wide range of architectures (including GPU).

### Search tree exploration prototype

When performing inexact mapping a recursive approach over a search tree can be employed [9]. This analysis depends on three factors. The first one is the current state of the backward search, each path from the root to any node of the tree represents a sequence of symbols that has lead to a different [ *k*,*l*] interval. The second one is the specific variability of the reference genome studied, on the initial tree levels only few symbols have been processed, so branches for all possible errors widely satisfy *k*≤*l* and grow uncontrollably. The third one is the singularity of each read, which determines the minimum number of errors needed to map it.

We developed a search tree exploration algorithm that greatly reduces the tree growth during inexact search. It employs a faster iterative approach, using several lists to store partial results. These lists store previous results, next results to explore and final results. We named these lists *r* *l*_*p*_, *r* *l*_*n*_ and *r* *l*_*f*_ in the pseudo-code.

Algorithm 3 is a simplified prototype of the final approach. It lacks the bounding techniques described in next section, so its execution is not as efficient. We use it to explain the behaviour of the complete algorithm as it is based on the same subroutines: a selective **exact** search procedure that detects and annotates the positions where it is worth to study sequence errors and a conservative **branch** procedure with specific rules for genomic data.

The execution starts by adding a single partial result to the previous results list. This first single result contains the initial interval, no symbols of *W* analysed and an empty error list. After that, the **exact** and **branch** subroutines are executed alternatively, increasing the partial results stored in the previous and next lists. At the end, the last **exact** call returns the final results.



The **exact** subroutine (Algorithm 4) input variables are *inexact*, which indicates if it must perform an exact search or allow errors, and *last*, which indicates the last symbol to analyse (in this case the full string). It takes partial results from *r* *l*_*p*_ and analyses them, inserting in *r* *l*_*n*_ new partial results for each position requiring branches. These partial results denote other possible mappings in the reference that differ from the current read at the detected positions. In order to detect these positions we demonstrate the following condition.



Let [ *k* ^′^,*l* ^′^]←**search_iteration** ([ *k*,*l*],*a*) be two subsequent SA intervals in a forward search, where *a*=*W*[ *i*]. We define *r* *e* *s*=*l*−*k*+1 and *r* *e* *s* ^′^=*l* ^′^−*k* ^′^+1, these values indicate respectively the number of appearances of substrings *V*=*W*[ 0:*i*−1] and *V* ^′^=*W*[ 0:*i*] in the reference *X*. We demonstrate that for any read *W* and any BWT index *r* *e* *s* ^′^≤*r* *e* *s* is always true. If *V* appears *res* times in *X*, then *r* *e* *s* ^′^∈[ 0,*r* *e* *s*], because *V* is the prefix of *V* ^′^ (*V* ^′^=*V* *a*).

We observed that the number of potential results remains stable (*r* *e* *s*=*r* *e* *s* ^′^) and near its final value after several **search_iteration** (15 in *Drosophila Melanogaster* and 31 in *Homo Sapiens*). The positions with possible errors are the ones in which *r* *e* *s* ^′^<*r* *e* *s*, showing that the interval has lost reads that could be mapped allowing errors. This pruning is based on the current state of the search.

Section “Number of partial solutions” shows the *res* values of the substrings of “AGGATC” during a forward search against the reference “AGGAGC$”. The possible error branches should only be studied at positions 2 and 4 of the string, the substrings “AG” and “AGGA” where the values of *res* change. For substring “AG” this means that there is an alternative solution with “AGC” in the reference, instead of “AGG”. For substring “AGGA” the alternative solution is “AGGAG” instead of “AGGAT”. When *r* *e* *s*=−1 the string does not belong to the reference (*k*>*l*). When studying larger genomes, the pruning is not effective in the first iterations, but later on the values of *res* stabilise.

#### Number of partial solutions

Values of *res* during a forward search of string “AGGATC” against the reference “AGGAGC$”.



This technique also eliminates redundant results, i.e. when mapping “TGGGGGA” into “…TGGGGA…” we would obtain five different results, one for each possible deletion of any of the ‘G’ nucleotides. Now, the *r* *e* *s* ^′^<*r* *e* *s* condition is only true in the last ‘G’, obtaining a single deletion as result.

The **branch** subroutine (Algorithm 5) extracts partial results from *r* *l*_*n*_, generates new branches by studying the outcomes of adding different errors at *r*.*p* *o* *s* *i* *t* *i* *o* *n* and stores the valid ramifications in *r* *l*_*p*_. The notation *p*.{*D*,*I*(*b*),*M*(*b*)}:*r*.*e* *r* indicates that we add a deletion, insertion or mismatch with symbol *b* in position *p* to the list of errors of the current partial result *r*.*e* *r*. Unlike this approach, algorithms that use backward search only for seeding do not need to obtain alignment information before the local alignment phase.

As the branches that do not satisfy *k*≤*l* are eliminated, this pruning depends on the variability of the reference genome.



All the pairs of consecutive errors are analysed in order to further reduce the growth of the search tree, forbidding those equivalent to a single error. For simplicity, these restrictions are not described in the pseudo-code:

- Pairs of consecutive insertions and deletions (I-D or D-I) are not allowed. Inserting a nucleotide and immediately removing it has no significance. This rule avoids *indel* chains like I-D-I-D-I. Also, I-I-I-D-D chains are avoided, as I-M-M chains are equivalent.
- A mismatch after an insertion is not allowed if the original nucleotide in the mismatch position is the same as the nucleotide of the insertion. In such cases I-M is equivalent to I.
- A mismatch after a deletion is not allowed if the nucleotide of the mismatch is the same as the nucleotide eliminated by the deletion. In such cases D-M is equivalent to D.
- An insertion after a mismatch is not allowed if the nucleotide of the insertion is the same as the original nucleotide in the mismatch position. In such cases M-I is equivalent to I.
- A deletion after a mismatch is not allowed if deleted nucleotide is the same as the nucleotide of the mismatch. In such cases M-D is equivalent to D.

After applying these rules the growth of the spanning tree is halved. In addition, inexact searches with up to 2 errors will not produce repeated results.

### Search tree exploration complete algorithm

**Figure 1:**
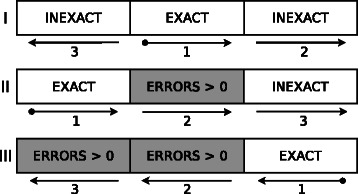
**Complete inexact search algorithm.** Example for 2 errors, from top to down steps I, II and III.

The bounding strategies of **branch** and **exact** are based on *k*≤*l* and *r* *e* *s* ^′^<*r* *e* *s* conditions, being not effective with few symbols analysed. The final algorithm depicted in Figure 1 and Algorithm 6 solves this issue with no penalty in sensitivity.

For the complete algorithm to work we need backward and forward versions of the **branch** and **exact** subroutines (**branchB** and **branchF**) and a new function to change the direction of the search in the partial results that reach the end of the read (**change_direction**).

The complete algorithm is based on the work presented in [23], with improvements to avoid repeated computations and extended support for more than two errors with insertions, deletions and mismatches. We do not use bidirectional BWT, as it may not be so efficient with backward search methods based on SA that need a binary search in each iteration. Moreover, keeping track of the reverse and strand SA intervals also increases the number of memory writes when managing the partial results. Nevertheless, a bidirectional method would reduce the memory requirements of the algorithm.

In order to allow *e* errors we conceptually divide the read in *e*+1 segments and perform *e*+1 steps. Figure 1 shows an example for two errors (*e*=2): the steps are in roman numerals, the arrows indicate the direction of the analysis in each segment and the arrow numbers the order in which the segments are analysed.

The first segment of each step is analysed using an exact search, if the segment is not found in the reference the whole step is skipped. After this initial exact analysis the pruning methods are effective and the remaining segments can be analysed with inexact search. Due to this, the number of errors allowed by the algorithm depends on the length of the read and the minimum segment size (31 for human genome and 15 for Drosophila Melanogaster, *segsize* in Algorithm 6). Also, it is worth to mention that these exact segments could be reused later as seeds to find local alignment regions.

In step I (Algorithm 6), after analysing block of arrow 2 the direction of the search is changed. As we have the SA and ISA of the reference and its reverse in memory we can use the **change_direction** subroutine to change the direction of the search. In practical use the size of the SA intervals when changing direction is very small (almost always equal to 1), so this computation does not affect the performance. Notice that at each step the starting block and the direction maximises the number of symbols analysed before a direction change.

In step II and III, during the analysis of partial results in the blocks marked with *e* *r* *r* *o* *r* *s*>0 the next partial results must contain at least one error within the block, due to this when the analysis reaches the end of the block the partial result with no errors in that segment is not added to the next results list. The logic of this bounding can be added to the **exact** subroutine with an extra condition in line 15 of Algorithm 4 to discard exact segments. This behaviour avoids repeated computation as the segments with no errors where already checked in the exact blocks of previous steps. Notice that the order of the steps maximises the appearances of *e* *r* *r* *o* *r* *s*>0 blocks immediately after the exact block.



The tree exploration is a combination of *breadth first search* (BFS) and *depth first search* (DFS), being a bit more complex than the pseudo-code in Algorithm 6. The initial levels are explored using BFS while the last ramification, where many partial results are discarded and only a few final results are kept, is explored using DFS. This avoids a great amount of memory writes in the partial results lists. Using DFS in the initial levels has little effect in the overall performance.

The last bounding technique depends on the uniqueness of the read and is based on what was presented in [9], but adapted to the new algorithm and the direction changes. Before each step of the complete algorithm, a vector *D* with the same size of the read is built. The *D* vectors contain an approximation of the number of errors needed to map the read at each position, based on the sub-strings of *X* present in *W*.

In Algorithm 7, vector *D* for step I of the complete algorithm is obtained. It receives as parameters the read *W* and the *start* and *end* positions of the exact segment of the current step, returning vector *D*. The values of *D* for the exact segment are already calculated (*D*[ *s* *t* *a* *r* *t*…*e* *n* *d*]=0). The direction of the calculation is changed in the second loop. In the last loop the values are adjusted as we calculate vector D in the same direction of the search, which benefits caching. The condition “is not a substring” is implemented using a **search_iteration**.

In order to implement this bounding, an extra condition at line 11 of Algorithm 4 must check the current number of errors against the value of *D*[ *i*].



### Experiments configuration

All the executions have been performed in a PC with an Intel(R) Core(TM) i7-3930 K CPU running at 3.20GHz speed, 64GB of DDR3 1066 MHz RAM and a Raid 0 of two OCZ-VERTEX4 SSD drives. The operative system is Ubuntu Linux 14.04 64 bit. Compiler is gcc 4.8.2. All the tests in the results section have been launched sequentially, using a single execution thread with no parallelism involved.

The same index has been generated for all tools: Ensembl 68 human genome built upon GRCh37. The program dwgsim 0.1.8 from SAMtools was used to simulate two datasets of 2 million high quality Illumina reads. One dataset contains 250 bps reads while the other contains 400 bps reads. The datasets contain reads with a maximum of 2 N’s and 0.1% of mutations with 10% indels.

## Results and Discussion

### Comparison with other FM-Index only algorithms

As we stated before, our algorithm is not intended as a full sequence mapper, only a preprocessing step for modern sequence mappers. The purpose of this study is to provide a fair comparison against similar algorithms based only on FM-Index backward search, performing the experiments under the same input, execution arguments and system environment.

We only found similar implementations to our algorithm in Bowtie 1, SOAP 2 and BWA-backtrack. Comparing with these tools gives an idea of the impact in the performance of the improvements described in this paper.

The tools have been run with -a option in order to find all the possible mappings of each read; except BWA-backtrack, which focuses on finding the best mapping for each read.

Our algorithm has been run with a stack size of 50.000 partial results, big enough to deal with all the partial results without discarding any read locations. We also conducted tests with an stack size of 500 partial results, which increases performance without significant mapping location loss. The minimum segment size needed to deal with the human genome variability is 31 nucleotides, allowing up to 7 errors with the 250 bps dataset.

Results in Table 1 show that our algorithm with a stack size of 50000 achieves a 8 × speed-up over Bowtie 1 when aligning with 3 errors and a 7 × speed-up over SOAP2 when aligning with 2 errors. Our algorithm can map with 5 errors in less time than Bowtie 1 with 3 errors. Execution times for the exact mapping case with no errors were similar for all the algorithms studied except BWA-backtrack. In general our algorithm is faster than the other approaches. This difference increases with the number of errors.

**Table 1:** **Results for soap 2, Bowtie 1, BWA-backtrack and the new algorithm**

	**Time**	**% Found**	**Locations**
**Soap 2**			
0 errors	25 s	0.51%	11025
1 errors	41 s	3.22%	71365
2 errors	6 m 34 s	10.25%	243599
**Bowtie 1**			
0 errors	24 s	0.51%	11025
1 errors	51 s	3.22%	71365
2 errors	4 m 58 s	10.25%	243599
3 errors	12 m 13 s	22.53%	594626
**BWA-backtrack**			
0 errors	1 m 17 s	0.51%	
1 errors	1 m 19 s	3.22%	
2 errors	1 m 30 s	10.29%	
3 errors	2 m 2 s	22.65%	
4 errors	4 m 35 s	38.73%	
5 errors	16 m 28 s	55.44%	
6 errors	60 m 17 s	69.78%	
**GRyCAP-BWT**	*Stack size 50000*		
0 errors	21 s	0.51%	11025
1 errors	35 s	3.22%	72546
2 errors	52 s	10.29%	253479
3 errors	1 m 28 s	22.66%	644415
4 errors	2 m 45 s	38.74%	1188595
5 errors	7 m 24 s	55.46%	1820725
6 errors	23 m 45 s	69.78%	2830556
**GRyCAP-BWT**	*Stack size 500*		
0 errors	21 s	0.51%	11025
1 errors	31 s	3.22%	72546
2 errors	51 s	10.29%	246841
3 errors	1 m 21 s	22.60%	515399
4 errors	2 m 1 s	38.44%	881503
5 errors	3 m 3 s	54.46%	1296682
6 errors	4 m 40 s	67.46%	1716369
7 errors	7 m 12 s	76.19%	2135443

The dataset contains 2 million 250 bps reads.

Table 1 also shows the percentage of reads found and the total mapping locations. The percentage represents if a read is found at least once in the reference, while in the mapping locations a read may appear several times. These values demonstrate that our algorithm performs an equivalent computation to Bowtie 1 and Soap 2, finding a similar amount of reads and mapping locations when allowing the same number of errors. Compared with BWA-backtrack we find the same percentage of reads.

Regarding the experiments with an stack size of 500 elements, our algorithm is 13 × faster than BWA-backtrack when mapping with 6 errors. Results show that limiting the stack size to 500 elements has little effect in the percentage of reads found (up to 2% less with 6 errors). However, the execution time is greatly decreased as the number of total mapping locations is reduced. As the mapping locations found with a small stack size are the ones with less errors, this approach is very useful for finding the best alignments.

During execution, our implementation has a memory footprint of 7GB, while Bowtie 1, SOAP 2 and BWA-backtrack consumed around 3 GB of RAM. This difference is because our algorithm is using indices for both forward and backward search. Although our algorithm requires more memory, it is still able to run in current desktop computers.

### Preprocessing step for modern aligners

The purpose of the experiments in this section is to quantify how well our algorithm would perform as a preprocessing step for modern sequence mappers, concretely Bowtie 2 v2.2.3 and BWA-MEM v0.7.10. Such mappers combine backward search seeding with local alignment algorithms based on dynamic programming.

We compare the execution times of these modern sequence mappers alone against a pipeline that launches our algorithm, annotates the reads found when it has finished and then launches one of the mappers to find the remaining reads. Using this configuration the sensitivity is not modified, finding the same amount of reads.

Bowtie 2 and BWA-MEM have been run with its default execution parameters, finding in most cases the best occurrence of each read. Our algorithm has been run with a similar configuration by limiting the size of the partial result lists to 500 elements.

**Figure 2:**
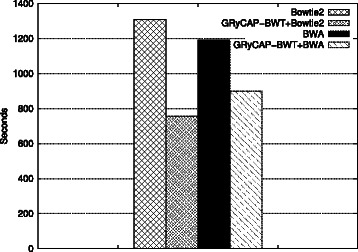
**BWT and SW tools.** 2 Million 250 bps reads. Execution times comparing the new algorithm, the modern mappers and the combination of both.

Figure 2 shows execution times for mapping the whole 250 bps dataset. Bowtie 2 execution took 21 m 47 s and BWA-MEM execution took 19 m 52s. For the combined alignment our algorithm was executed allowing up to 5 errors, finding 54.46% of the reads in 3 m 2s. The remaining reads where feed to Bowtie 2 and BWA-MEM resulting in a total execution time of 12 m 37 s and 15 m 1 s respectively. This combined approach improves total alignment time by 42% for Bowtie 2 and 25% for BWA-MEM. Bowtie 2 found 94.26% of the reads and BWA-MEM found 94.48%, the same amount of reads where found when using our algorithm as a preprocessing step.

**Figure 3:**
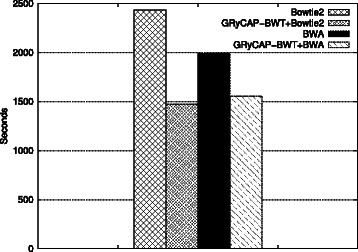
**BWT and SW tools.** 2 Million 400 bps reads. Execution times comparing the new algorithm, the modern mappers and the combination of both.

Figure 3 shows execution times for the 400 bps dataset, Bowtie 2 took 40 m 35 s and BWA-MEM took 33 m 09s. Our algorithm was executed allowing up to 9 errors, finding 62.21% of the reads in 9 m 24s. The combined approach with Bowtie 2 took 24 m 33 s (40% faster) and with BWA-MEM took 25 m 57 s (21% faster). Bowtie 2 found 94.46% of the reads and BWA-MEM found 94.48%, the same amount of reads where found when using our algorithm as a preprocessing step.

Interestingly, these results reveal a greater performance improvement when combining our algorithm with Bowtie 2. In these experiments, BWA-MEM is faster than Bowtie 2 for aligning all reads. However, when using the preprocessing proposed in this paper Bowtie 2 becomes faster.

### Comparison between BWT and *csalib* runtimes

When dealing with big genomes, the size of the SA may be greater than the memory capacity of the machine. In this section we compare the speed of our algorithm using the BWT and the *csalib* out-of-core runtimes. This library is developed at the National Institute of Informatics in Tokyo (Japan) [29].

The BWT runtime uses about 7GB of RAM for the human genome index, while the *csalib* runtime does not load the index into memory. With *csalib* the index is directly read from disk using *mmap*, needing only a few hundreds of Megabytes of RAM to map reads.

**Figure 4:**
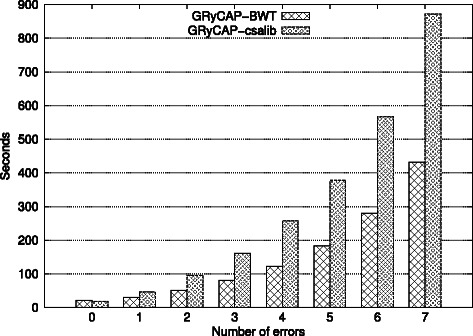
**BWT and csalib runtimes.** 2 Million 250 bps reads. Execution times from 0 to 7 errors with stack size 500.

Figure 4 shows a 100% increase in execution time when using the *csalib* runtime, across all error configurations. When using csalib the asymptotic cost of the algorithm is not modified, demonstrating the viability of this approach with currently affordable SSD disk configurations.

### Asymptotic analysis

In this section we analyse the asymptotic cost of the algorithm. We also compare it with the other approaches studied.

The function that defines the growth of a search tree is $\mathcal {O}(k^{e})$, where *k* is the branching factor and *e* is the depth of the tree. The branching factor of a search tree is obtained by dividing the total number of branches by the number of nodes with descendants.

In a trivial algorithm a full search tree is spawn at every position of the search string. The branching factor of the tree depends on the alternatives available: match, 4 mismatches and 4 insertions (one for each symbol). So, the asymptotic cost for an algorithm without optimisations is $\mathcal {O}(9^{e})$, where the depth of the tree *e* is the number of errors allowed during the search.

Bowtie 1 and SOAP2 do not support indels, reducing the cost to $\mathcal {O}(5^{e})$. This cost reduction comes at the expense of exploring less options and finding less mapping locations.

We employ the pruning techniques described previously to reduce the tree growth. The effectiveness of these techniques depends on the read analysed and the variability of the genome. Also, the worst case happens when few symbols of *W* are analysed, because all the branches exist in the reference. In practice, we perform exact search on the first symbols allowing errors later (Figure 1). This way the number of branches is reduced.

Due to these variant factors, we studied the average branching factor of the search tree experimentally. We have randomly chosen 1000 reads from the 2 Million 250 bps dataset. We aligned these reads with our algorithm allowing different number of errors in order to obtain the average branching factor. This parameter is an estimation of the growth of the tree, obtaining an asymptotic cost of $\mathcal {O}(2.53^{e})$.

This is a great improvement compared with an algorithm without optimisations ($\mathcal {O}(9^{e})$), while still allowing errors in any position of the read (including indels).

## Conclusions

Improving previous research [9,23], we have developed a fast backward search algorithm for inexact sequence mapping (including mismatches, insertions and deletions). This algorithm is up to 13 × faster than similar algorithms implemented in Bowtie, SOAP2 and BWA-backtrack. This impressive speed-up allows to handle more errors than before within a reasonable amount of time.

The proposed algorithm has been validated as a mapping preprocessing step, reducing the number of reads to align by 55%. This improves execution time of Bowtie 2 and BWA-MEM by about 40% and 20% respectively, mapping the same amount of reads in the same positions. The practical limit of errors allowed in this preprocessing appears when the seeding and local alignment becomes faster. We obtain good results with both 250 bps and 400 bps datasets, allowing up to 5 and 9 errors respectively.

Our implementation is built upon a modular architecture, being compatible with different backward search techniques. We tested an out-of-core implementation of the FM-Index provided by *csalib* library, obtaining reasonable execution times, showing the viability of cost-effective secondary memory configurations.

As future work, the computation of the exact segments done by the algorithm could be reused in the seeding phase, improving even further the speed of the overall process.

Furthermore, the mapping locations obtained by the algorithm must processed taking into account quality scores and gap penalties to match the criteria of the mapping tool on which is integrated. This post-process will not affect the logic nor the performance of the proposed algorithm.

The source code of our implementation is available under the LGPL license and could be easily integrated in current mapping software. The source code, binaries and datasets of the experiments can be found at http://josator.github.io/gnu-bwt-aligner/.

## Abbreviations

NGS: Next generation sequencing
SW: Smith-waterman
HMM: Hidden Markov models
FM-Index: Ferragina and Manzini index
BWT: Burrows wheeler transform
SA: Suffix array
ISA: Inverse suffix array
BFS: Breadth-first search DFS: Depth-first search
